# Hot flashes are not predictive for serum concentrations of tamoxifen and its metabolites

**DOI:** 10.1186/1471-2407-13-612

**Published:** 2013-12-28

**Authors:** Nynke GL Jager, Rutger HT Koornstra, Andrew D Vincent, Ron HN van Schaik, Alwin DR Huitema, Tiny M Korse, Jan HM Schellens, Sabine C Linn, Jos H Beijnen

**Affiliations:** 1 Department of Pharmacy & Pharmacology, Slotervaart Hospital/The Netherlands Cancer Institute, Louwesweg 6, 1066 EC Amsterdam, The Netherlands; 2 Department of Molecular Pathology, The Netherlands Cancer Institute, Plesmanlaan 121, 1066 CX Amsterdam, The Netherlands; 3 Department of Biometrics, The Netherlands Cancer Institute, Plesmanlaan 121, 1066 CX Amsterdam, The Netherlands; 4 Department of Clinical Chemistry, Erasmus University Medical Centre, ‘s Gravendijkwal 230, 3015 CE Rotterdam, the Netherlands; 5 Department of Clinical Chemistry, The Netherlands Cancer Institute, Plesmanlaan 121, 1066 CX Amsterdam, The Netherlands; 6 Department of Clinical Pharmacology, The Netherlands Cancer Institute, Plesmanlaan 121, 1066 CX Amsterdam, The Netherlands; 7 Department of Pharmaceutical Sciences, Faculty of Science, Division of Pharmacoepidemiology & Clinical Pharmacology, Utrecht University, 3508 TB Utrecht, The Netherlands; 8 Department of Medical Oncology, The Netherlands Cancer Institute, Amsterdam, The Netherlands

**Keywords:** Endoxifen, Tamoxifen, Hot flashes, Estrogen levels, CYP2D6, Breast cancer

## Abstract

**Background:**

Tamoxifen has dramatically reduced the recurrence and mortality rate of estrogen receptor positive breast cancer. However, the efficacy of tamoxifen varies between individuals and 40% of patients will have a recurrence despite adjuvant tamoxifen treatment. Factors that predict tamoxifen efficacy would be helpful for optimizing treatment. Serum concentrations of the active metabolite, endoxifen, may be positively related to treatment outcome. In addition, hot flashes are suggested to be positively associated with tamoxifen treatment outcome.

**Methods:**

We investigated in a series of 109 patients whether the frequency and severity of hot flashes were related to concentrations of tamoxifen and its metabolites. A serum sample of all patients was analyzed for the concentration of tamoxifen, N-desmethyltamoxifen, endoxifen and 4-hydroxytamoxifen, as well as for estradiol concentrations and several single nucleotide polymorphisms in CYP2D6. Additionally, these patients completed a questionnaire concerning biometric data and treatment side effects.

**Results:**

We found no evidence supporting an association between concentrations of tamoxifen or metabolites and either the frequency or severity of hot flashes in the covariate unadjusted analyses. However, including interactions with menopausal status and pre-treatment hot flash (PTHF) history indicated that post-menopausal women with PTHF experienced an increasing frequency of hot flashes with increasing serum concentrations of tamoxifen and its metabolites. This finding was not altered when adjusting for potential confounding factors (duration of tamoxifen treatment, CYP2D6 phenotype, estradiol serum concentration, age and body mass index). In addition we observed a positive association between body mass index and both hot flash frequency (p = 0.04) and severity (p < 0.0001). We also observed that patients with lower estradiol levels reported more severe hot flashes (p = 0.02).

**Conclusions:**

No univariate associations were observed between concentrations of active tamoxifen metabolites and either the frequency or severity of hot flashes during treatment. However, the frequency of hot flashes may be exacerbated by higher serum concentrations of tamoxifen and its metabolites in post-menopausal women with a history of hot flashes prior to tamoxifen treatment.

## Background

For over 30 years tamoxifen, a selective estrogen receptor (ER) modulator, has been the standard treatment for estrogen receptor positive breast cancer patients, in both the adjuvant and metastatic setting. Tamoxifen has dramatically reduced the recurrence and mortality rate for patients with ER + breast cancer [1]. However, as many as 40% of patients receiving adjuvant tamoxifen and almost all patients with metastatic disease eventually relapse and die from the disease [2]. Due to this high percentage of patients with an apparent lack of benefit, identification of early predictors of outcome of tamoxifen treatment may be helpful in the optimization of the treatment [3].

Tamoxifen itself is considered to be a prodrug that is converted into many metabolites. The metabolites with the highest therapeutic activity are 4-hydroxytamoxifen and N-desmethyl-4-hydroxytamoxifen (endoxifen), binding 100-fold more potent to the ER than tamoxifen itself [4]. The antiestrogenic activities of endoxifen and 4-hydroxytamoxifen are similar, although endoxifen, unlike 4-hydroxytamoxifen, also inhibits aromatase and is present at higher steady state concentrations in patients than 4-hydroxytamoxifen [4-7]. Recently, Madlensky *et al.* reported that low endoxifen levels are associated with worse outcome after tamoxifen treatment, suggesting that there is a minimum threshold serum level of endoxifen that when exceeded lowers the recurrence rate [8]. However, assays for routine measurement of concentrations of tamoxifen and its metabolites are not generally available in daily practice. Therefore, the quest for other biomarkers for treatment efficacy is still ongoing.

Tamoxifen is metabolized by cytochrome P450 (CYP) enzymes, in which the formation of endoxifen predominantly depends on CYP2D6. Inactivating genetic polymorphisms in CYP2D6 have been associated with lower endoxifen levels [9-11] and consequently CYP2D6 genotype has been suggested as a potentially useful marker for the prediction of treatment outcome. Recently, the ATAC and the BIG1-98 studies concluded that genetic variants of CYP2D6 are not predictive for outcome in tamoxifen-treated patients [12,13], although the validity of these findings has been questioned [14].

The occurrence of side effects, such as hot flashes, is a potential biomarker for treatment outcome, analogous to what has been described with EGFR inhibitors and skin-toxicity [15]. It is known that breast cancer patients treated with tamoxifen suffer more frequently from hot flashes, compared to placebo-treated breast cancer patients [16]. The severity of hot flashes is suggested to increase during the first three months of tamoxifen treatment, followed by a plateau or even a decrease for the duration of treatment [17,18].

Mortimer *et al.* showed that the occurrence of hot flashes is positively related to outcome after tamoxifen treatment [19]. Cuzick *et al.* investigated whether the occurrence of treatment-related symptoms (vasomotor symptoms or joint symptoms) is associated with breast cancer recurrence. They found a trend that patients using tamoxifen who experienced newly emergent vasomotor symptoms (e.g. hot flushes, night sweats and cold sweats) had a lower recurrence rate, although these results were not statistically significant [20].

Recently, Lorizio *et al.* reported that the serum concentration of endoxifen is positively associated with the probability of reporting any side effect from tamoxifen (hot flashes, vaginal dryness, sleep problems, weight gain, and depression, irritability or mood swings combining all side effects and grades). When focusing on hot flashes only, this association was not statistically significant. Irvin *et al.* performed a genotyped tamoxifen dose-escalation study and found no correlation between endoxifen concentrations and the extent to which patients were bothered by hot flashes, neither at baseline nor at four months after dose escalation [10].

In order to clarify whether there is an association between concentrations of tamoxifen and its main metabolites and either frequency or severity of hot flashes, we investigated a series of 109 patients treated with tamoxifen, taking into account potentially influencing factors such as menopausal status, pre-treatment hot flashes, duration of tamoxifen treatment, CYP2D6 phenotype, estradiol serum concentrations, age and body mass index (BMI).

## Methods

Patients, both pre- and postmenopausal, who used tamoxifen for at least two months at the moment serum concentrations of tamoxifen and metabolites were determined as part of routine clinical care were eligible for this study. Retrospectively, these patients were asked whether they would be willing to complete a single, short questionnaire (Additional file 1) concerning biometric data and the side effects they had experienced. The questionnaire was sent to the patients along with an informative letter, stating the goal of this study and explicitly giving the patients the option to opt-out, by returning the questionnaire without filling it out. By this questionnaire, patients were asked if they had been experiencing hot flashes prior to beginning tamoxifen treatment, and also if they experienced hot flashes during tamoxifen treatment (around the time the blood sample was drawn). In both cases the patients were asked to record the frequency of the flashes per week and the average severity of the experienced hot flashes (severity categories: mild, <5 minute duration; moderate, 5 to 15 minute duration; severe, 15 to 20 minute duration; very severe, >20 minute duration). These definitions were based on the methodology and instruments for conducting hot flash studies [21,22].

We performed this observational study with a simple, single questionnaire according to the national act on Ethics Committees (Dutch Act on medical research involving humans, February 26, 1998) and in compliance with Good Clinical Practice guidelines [23]. As a further interpretation of these GCP guidelines there is the “code of conduct of Human Tissue and Medical Research: Code of conduct for responsible use (2011)” by the Federa (http://www.federa.org/codes-conduct). In this code of conduct is stated that anonymous left-over body material may be used in observational clinical trials without explicit consent of the individual patients.

### Serum sample handling and determination of tamoxifen and metabolites

The serum samples were collected in serum gel tubes and stored at -70°C for some weeks, in order to analyze more patient samples during one HPLC-MS analysis. Patient samples, calibration standards and quality control samples were handled according to the method described by Teunissen *et al.*[24]. The liquid chromatography – tandem mass spectrometry (LC-MS/MS) method developed by Teunissen *et al.*[24] was slightly modified and used for the determination of tamoxifen (5 to 500 ng/mL), N-desmethyltamoxifen (10 to 1000 ng/mL), *(E)*-endoxifen (1 to 100 ng/mL), *(Z)*-endoxifen (1 to 100 ng/mL), N-desmethyl-4′-hydroxytamoxifen (1 to 100 ng/mL), 4-hydroxytamoxifen (0.4 to 40 ng/mL) and 4′-hydroxytamoxifen (0.4 to 40 ng/mL). Detection was performed on a triple-quadrupole MS/MS detector with an electrospray ionization source (API4000, AB Sciex, Foster City, USA) operating in the positive ion mode. A partial validation was executed and all requirements for acceptance, as defined in the FDA and EMA guidelines on bioanalytical method validation [25,26] were fulfilled.

### Genotyping and predicted phenotype

DNA was isolated from 200 μL serum that was left over from the tamoxifen and metabolite analysis, using the MagNA Pure LC Total Nucleic Acid Isolation Kit I and the automated MagNA PureTM LC system (Roche Diagnostics, Mannheim, Germany) according to the manufacturer’s manual.

Genotyping was performed according to Standard Operating Procedures, using assays that were validated by direct sequencing. In each run, positive and negative controls were included. All patients were genotyped for CYP2D6*3, *4, *6 and *41 variant alleles, which will identify 95% of CYP2D6 poor metabolizers (PMs) using Taqman allelic discrimination assays with primers and probes designed by Applied Biosystems (Carlsbad, California, USA), as described earlier [27]. Polymerase chain reactions (PCR) were carried out in a reaction volume of 10 μl, containing 1 ng genomic DNA. The thermal profile consisted of an initial denaturation step at 95°C for 15 minutes, followed by 40 cycles of denaturation at 92°C for 15 seconds and 1 minute at 60°C for annealing and extension. Genotypes were scored through measuring allele-specific fluorescence using the SDS 2.2.2 software for allelic discrimination (Applied Biosystems).

On the basis of CYP2D6 genotype patients were classified into three predicted phenotype groups. Patients without nonfunctional alleles (CYP2D6*3, *4 or *6) were defined as extensive metabolizers (EMs). Intermediate metabolizers (IMs) consisted of patients that (i) carry CYP2D6*41 alleles either homozygous or in combination with a nonfunctional allele or (ii) were heterozygous for the CYP2D6*3, *4, *6 allele (*3/wt, *4/wt or *6/wt). Patients were classified as PM in case of two nonfunctional alleles (CYP2D6*3/*3, *3/*4 or *4/*4).

### Estradiol concentration

The estradiol concentration was measured in the left over serum sample on a Modular Analytics E170 immunoassay analyzer, using the electrochemiluminescence technique (Roche Diagnostics), routinely used in the Netherlands Cancer Institute.

### Statistical methods

The relation between hot flashes and several factors was investigated, where the serum concentrations of tamoxifen and three of its main metabolites (N-desmethyltamoxifen, endoxifen and 4-hydroxytamoxifen) were considered of primary interest. In addition there were seven secondary factors that may have a potential role confounding role: menopausal status, a history of hot flashes prior to tamoxifen treatment, duration of tamoxifen treatment, estradiol serum concentration, age, BMI and CYP2D6 predicted phenotype. The association between all factors and menopausal status was assessed using Mann–Whitney-Wilcox, Fisher exact and linear-by-linear tests as appropriate. Spearman’s rho was used to assess pairwise covariate associations between the four primary factors (tamoxifen and metabolite serum concentrations), age, BMI and estradiol concentration. Linear by linear trend tests were used to assess the association between CYP2D6 phenotype and the four primary factors. Kruskal-Wallis tests was used to determine if the four factors differed due to menopausal status and pre-treatment hot flash history. The association between reported hot flash frequency and both primary and secondary factors was assessed using over-dispersed Poisson models, both unadjusted (univariable) and multivariable regressions. Similarly, the association between all factors and the severity of hot flashes was assessed using proportional-odds ordinal regressions. It was assumed that these associations may be influenced by menopausal status and the occurrence of pre-tamoxifen treatment hot flashes (PTHF). Due to the small number of pre-menopausal women reporting PTHF the influence of menopausal status and PTHF was assessed via pair-wise interactions with a three level menopausal and pre-treatment hot flash status variable (pre-menopausal versus post-menopausal & PTHF versus post-menopausal & no PTHF). In the multivariable analyses, estradiol concentrations were log transformed and missing estradiol and CYP2D6 values due to insufficient material were imputed with population medians. Due to the large number of individuals missing for the CYP2D6 assessments, sensitivity analyses were performed; once with these individuals imputed as poor-intermediate metabolizers and once excluding these individuals. For samples with an estradiol concentration level below the lower limit of quantitation (43 pmol/L), half of the lower limit of detection (21.5 pmol/L) was imputed. The level of significance for all tests was set at 0.05. The analysis was performed using the R (v3.0.1) using package *MASS* for ordinal regression and *coin* for linear by linear tests (http://cran.r-project.org/).

## Results

### Cohort

Between July 2008 and December 2011 serum samples from 165 patients treated with tamoxifen at the Netherlands Cancer Institute, Amsterdam, the Netherlands were obtained and analyzed for tamoxifen and metabolite concentrations. These 165 patients received the questionnaire. 33 patients did not respond to the questionnaire that was sent and 13 patients returned the reply form empty, thereby choosing the option to opt-out and not participate in this study. In total, 119 patients returned a filled out questionnaire, of which 115 forms were correctly completed. Six patients were excluded for the following reasons: one patient had an uncertain menopausal status at the moment of blood sampling; one patient was taking medication to relieve menopausal complaints; it turned out that two patients used tamoxifen less than two months at the moment of blood sampling and two patients used tamoxifen for distant metastases for an exceptionally long time (over 6 years). In total, 109 patients (all female, age mean (range) 51 years (22–76)) were enrolled in the study. The patients were divided into two groups, based on menopausal status. Table 1 presents an overview of patient characteristics.

**Table 1:** Patient characteristics

**Characteristics**	**Total cohort**	**Pre-menopausal**	**Post-menopausal**	**p-value**
	**n = 109**	**n = 56**	**n = 53**	
	***n (%)***	***n (%)***	***n (%)***	
Median age at assessment (years)	51	45	58	<0.0001
Range	22 - 76	22-54	40 - 76	
Median Body Mass Index	24	24	24	0.44
Range	17 - 43	17 - 34	18 - 29	
T-status (TNM)				0.73
T1	48 (44%)	23 (41%)	25 (47%)	
T2	27 (25%)	16 (29%)	11 (21%)	
T3	2 (1.8%)	1 (1.8%)	1 (1.9%)	
Unknown	32 (29%)	16 (29%)	16 (30%)	
N-status (TNM)				1.00
N0	45 (41%)	24 (43%)	21 (40%)	
N+	49 (45%)	26 (46%)	23 (43%)	
Unknown	15 (14%)	6 (11%)	9 (17%)	
AJCC stage (7^th^ ed.)				0.87
Stage I	26 (24%)	14 (25%)	12 (23%)	
Stage IIa	32 (29%)	16 (29%)	16 (30%)	
Stage IIb	7 (6%)	5 (9%)	2 (4%)	
Stage IIIa	9 (8%)	4 (7%)	5 (9%)	
Stage IIIb	0	0	0	
Stage IIIc	6 (6%)	3 (5%)	3 (6%)	
Unknown	29 (27%)	14 (25%)	15 (28%)	
Estrogen receptor				NA
Positive	93 (85%)	50 (89%)	43 (81%)	
Unknown	16 (15%)	6 (11%)	10 (19%)	
Progesterone receptor				1.00
Positive	10 (9%)	5 (9%)	5 (9%)	
Negative	83 (76%)	45 (80%)	38 (72%)	
Unknown	16 (15%)	6 (11%)	10 (19%)	
HER2 status				1.00
Positive	38 (35%)	20 (36%)	18 (34%)	
Negative	55 (50%)	30 (54%)	25 (47%)	
Unknown	16 (15%)	6 (11%)	10 (19%)	
Median duration of treatment (months)	9	9	9	0.73
Range	2 - 70	2 - 59	3 - 70	
Tamoxifen (daily dose)				0.17
10 mg	1 (1%)	1 (2%)	0 (0%)	
20 mg	102 (94%)	50 (89%)	52 (98%)	
40 mg	6 (6%)	5 (9%)	1 (2%)	

T-status, Tumor status, N-status, Lymph node status, HER2, Human Epidermal growth factor Receptor 2.

Table 2 shows that the serum concentrations of tamoxifen and its metabolites were not significantly different between pre- and postmenopausal patients. A total of 92 patients (84%) reported experiencing hot flashes during tamoxifen treatment, with considerable variation in reported hot flash severity. Of patients who reported experiencing no hot flashes before start of tamoxifen treatment, 65 (79%) reported developing hot flashes during treatment whereas all patients who reported experiencing hot flashes prior to starting tamoxifen treatment reported experiencing hot flashes during treatment. The frequency and severity of the reported hot flashes during tamoxifen treatment did not differ significantly between pre- and postmenopausal patients. For two patients, estradiol values were missing, due to an insufficient amount of input material. For 70 (64%) samples the analyzed estradiol concentration was below the lower limit of quantification (LLOQ, 43 pmol/L).

**Table 2:** Hot flash frequency and severity and pharmacological and biochemical parameters of study participants during treatment with tamoxifen

	**Total**	**Pre-menopausal**	**Post-menopausal**	**p-value**
	**n = 109**	**n = 56**	**n = 53**	
	***n (%)***	***n (%)***	***n (%)***	
Pre-treatment history of hot flashes				0.04
No	82 (75%)	47 (84%)	35 (66%)	
Yes	27 (25%)	9 (16%)	18 (34%)	
Median frequency of hot flashes per week	21	21	21	0.77
Range	0 - 168	0 - 168	0 - 168	
Average severity of hot flashes				0.56
None	17 (16%)	9 (16%)	8 (15%)	
Mild	22 (20%)	13 (23%)	9 (17%)	
Moderate	55 (50%)	26 (46%)	29 (55%)	
Severe	11 (10%)	7 (12%)	4 (8%)	
Very severe	4 (4%)	1 (2%)	3 (6%)	
Median tamoxifen (ng/mL)	95.4	93.8	97.9	0.61
Range	39.7 - 237	50.0 - 220	39.7 - 237	
Median N-desmethyltamoxifen (ng/mL)	181	177	187	0.82
Range	82.3 - 532	94.3 - 532	82.3 - 439	
Median endoxifen (ng/mL)	9.12	8.59	9.16	0.75
Range	1.73 – 22.6	1.73 – 20.3	2.14 – 22.6	
Median 4-hydroxytamoxifen (ng/mL)	1.69	1.77	1.43	0.91
Range	0.74 - 4.23	0.74 - 4.23	0.78 - 3.51	
Median estradiol (pmol/L)				0.06
<LLOQ*	70	33	37	
43.0 – 67.0	12	6	6	
67.0 – 361	14	8	6	
>361	11	9	2	
Missing	2	0	2	
CYP2D6 phenotype				0.66
Extensive metabolizer	54 (50%)	28 (50%)	26 (49%)	
Intermediate metabolizer	30 (28%)	19 (34%)	11 (21%)	
Poor metabolizer	5 (5%)	2 (4%)	3 (6%)	
Missing	20 (18%)	7 (12%)	13 (24%)	

*<LLOQ is below the minimal quantification limit.

### Genotyping

CYP2D6 genotype predicted phenotype was evaluable for 89 patients (81.7%). 5 (4.6%) patients were classified as poor metabolizers (PM), 30 (27.5%) as intermediate metabolizers (IM) and 54 (49.5%) as extensive metabolizers (EM) (see Table 2). For the other 20 patients (18.3%) the DNA quality was not sufficient to allow genotyping.

### Covariate associations

Spearman’s correlation coefficients indicated a positive association between tamoxifen and its three main metabolites and a negative association between age and estradiol levels (see Additional file 2).

In addition, linear by linear tests indicated associations between CYP2D6 predicted phenotype and endoxifen (p < 0.0001), N-desmethyltamoxifen (p = 0.009) and 4-hydroxytamoxifen serum concentrations (p = 0.05), but not tamoxifen concentrations (p = 0.65) (see Additional file 3). Kruskal-Wallis tests indicated no pairwise associations between the combined menopausal and PTHF status variable and tamoxifen nor its three metabolites.

### Associations with hot flashes

**Figure 1:**
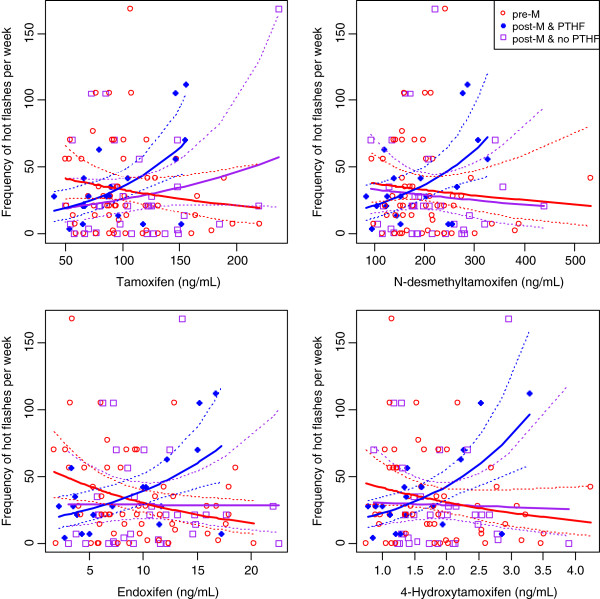
Hot-flash frequency plotted against tamoxifen and its metabolites, for pre- and post-menopausal women separately.

In the univariable Poisson and ordinal regressions no associations were found between the levels of tamoxifen, endoxifen or the two other metabolites and either the frequency or severity of hot flashes (see Table 3 and Additional file 4). When including a pairwise interaction with menopausal and PTHF status it was observed that the associations between tamoxifen and metabolite serum concentrations and the frequency of hot flashes were increasing for post-menopausal women with a pre-treatment history of hot flashes (see Table 3). Adjusting for potential confounding factors did not alter these results (Additional file 5; also see Additional file 6 for patient baseline characteristics by menopausal status and PTHF-status). Figure 1 presents the associations between serum concentrations of tamoxifen and its metabolites and patient-reported hot flash frequency in the menopausal and PTHF subgroups.

**Table 3:** Univariable Poisson regression associations with hot flash frequency (3A) and ordinal regression associations with hot flash severity (3B)

**3A**	**Univariable (N = 109)**			**Inter.**	**Pre-M. (N = 56)**			**Post-M & PTHF (N = 18)**			**Post-M & no PTHF (N = 35)**		
	**Coef**	**SE**	**p-value**	**p-value**	**Coef**	**SE**	**p-value**	**Coef**	**SE**	**p-value**	**Coef**	**SE**	**p-value**
**Tamoxifen**	0.002	0.0024	0.41	0.03	-0.0045	0.004	0.27	0.012	0.0038	0.01	0.0058	0.0044	0.19
**N-desmethyltamoxifen**	-0.00002	0.0013	0.99	0.13	-0.0013	0.002	0.50	0.0053	0.002	0.02	-0.0014	0.0031	0.66
**Endoxifen**	-0.015	0.022	0.50	0.01	-0.069	0.03	0.03	0.085	0.028	0.01	-0.0021	0.05	0.97
**4-Hydroxytamoxifen**	-0.05	0.14	0.73	0.03	-0.3	0.19	0.13	0.63	0.17	0.002	-0.056	0.37	0.88
**Post-M & PTHF v pre-M**	0.13	0.27	0.67										
**Post-M & no PTHF v pre-M**	-0.14	0.24											
**Age**	-0.0059	0.0098	0.55										
**Estradiol concentration**	-0.12	0.095	0.21										
**BMI**	0.048	0.023	0.04										
**Tamoxifen duration**	0.084	0.087	0.34										
**CYP2D6: EM versus I/PM**	-0.11	0.21	0.61										
**3B**	**Univariable (N = 109)**			**Inter.**	**Pre-M. (N = 56)**			**Post-M & PTHF (N = 18)**			**Post-M & no PTHF (N = 35)**		
	**Coef**	**SE**	**p-value**	**p-value**	**Coef**	**SE**	**p-value**	**Coef**	**SE**	**p-value**	**Coef**	**SE**	**p-value**
**Tamoxifen**	0.0026	0.0045	0.57	0.60	0.0026	0.0065	0.69	0.018	0.014	0.16	0.0019	0.0075	0.80
**N-desmethyltamoxifen**	-0.00043	0.0022	0.85	0.30	-0.00048	0.003	0.88	0.01	0.0067	0.11	-0.0024	0.0039	0.53
**Endoxifen**	-0.013	0.039	0.73	0.72	-0.027	0.055	0.62	0.044	0.092	0.63	0.025	0.072	0.73
**4-Hydroxytamoxifen**	-0.20	0.25	0.43	0.64	-0.11	0.31	0.74	0.39	0.70	0.58	-0.36	0.52	0.48
**Post-M & PTHF v pre-M**	0.94	0.51	0.11										
**Post-M & no PTHF v pre-M**	-0.17	0.42											
**Age**	-0.018	0.017	0.31										
**Estradiol concentration**	-0.34	0.14	0.02										
**BMI**	0.19	0.048	<0.0001										
**Tamoxifen duration**	0.25	0.18	0.15										
**CYP2D6: EM versus I/PM**	0.0058	0.41	0.99										

Inter, Interaction; pre-M, Pre-menopausal patients; post-M, Post-menopausal patients; PTHF, Pre-treatment hot flashes; *v*, Versus; Coef, Coefficient; SE, Standard error; BMI, Body mass index; EM, Extensive metabolizers; I/PM, Intermediate to poor metabolizers.

For tamoxifen and its metabolites the test of interaction with menopausal and PTHF status, and the within-group associations are also reported.

Positive associations were found between BMI and both hot flash frequency (p = 0.04) and severity (p < 0.0001) (Table 3A). We also observed that pre-menopausal patients with lower estradiol levels reported more severe hot flashes (p = 0.02) (Table 3B). Both of these results remained significant in the multivariable analyses (Additional file 5). The sensitivity analyses indicated that the estimated coefficients were unaffected by the imputation of the missing CYP2D6 levels. While the tests for interaction remained significant when the missing data were imputed (both as poor-intermediate and as extensive metabolizers), these tests were non-significant in the analysis excluding missing values, possibly due to the 18% reduction in sample size.

CYP2D6 predicted phenotype was not associated with hot flash frequency (p = 0.61) nor hot flash severity (p = 0.99) (Table 3).

## Discussion

In this study we were unable to find evidence supporting the hypothesis that either frequency or severity of hot flashes are associated with higher levels of tamoxifen or any of its main metabolites during treatment in our entire cohort, consisting of both pre- and postmenopausal patients. No differences were detected in the frequency of reported hot flashes between pre- and post-menopausal women, however the association between concentrations of tamoxifen and its metabolites and patient-reported hot flash frequency appeared to be influenced by menopausal status and pre-treatment hot flash history.

Previously, Lorizio *et al.* have suggested that the endoxifen serum concentration was associated with increased risk of hot flashes, although this finding was not statistically significant [28]. Irvin *et al.* found no association between the extent to which patients were bothered by hot flashes and endoxifen concentration, neither at baseline, nor at four months after dose escalation [10]. We initiated this study to investigate the association of concentrations of tamoxifen and its main metabolites and both severity and frequency of hot flashes, taking potential confounding factors, such as menopausal status, pre-treatment hot flash history, duration of tamoxifen treatment, CYP2D6 phenotype, estradiol levels, age and BMI, into account. We could, however, find no evidence to support this hypothesis in the whole cohort. In the earlier mentioned BIG1-98 study, the authors also investigated hot flash incidence and the aggravation of hot flashes in the first two years of tamoxifen therapy. They found an association between CYP2D6 phenotype and tamoxifen-induced hot flashes (p = 0.02): both PM and IM phenotypes had an increased risk of tamoxifen-induced hot flashes compared with EM phenotype [13], contradictory to what was expected. Additionally, Sestak *et al.*[29] and Goetz *et al.*[30] reported that they were unable to detect an association between CYP2D6 phenotype and the occurrence of hot flashes. In this study we also found no evidence supporting the hypothesis that either hot flash frequency or severity is associated with CYP2D6 predicted phenotype, however genotyping data was missing in 18% of the cases. The large percentage of genotyping failures can be explained by the fact that DNA was isolated from serum, since this matrix was left over from the tamoxifen and metabolite analysis, which is a reproducible and validated method for genotyping in our lab, however the yield is low. Although the physiology of hot flashes, in both healthy women and women with breast cancer, remains unclear, it has been observed that healthy postmenopausal women who experience hot flashes have lower estradiol levels than women who do not experience hot flashes [31-34]. In our series, we correspondingly observed that patients, especially pre-menopausal patients, with lower estradiol levels reported more severe hot flashes.

Another physiological factor that may influence the occurrence of hot flashes in healthy women is body mass index (BMI), although this relationship is still a matter of debate. Some studies found a positive association [35], others a negative association [36,37] or no association [38]. In our series patients with higher BMIs reported suffering from more frequent and severe hot flashes.

Tamoxifen is metabolized into many different metabolites by cytochrome P450, the formation of endoxifen is mainly dependent on CYP2D6 activity. As with other studies [9-11], we were able to demonstrate a positive association between CYP2D6 activity and serum concentrations of active tamoxifen metabolites.

Our study has the following limitations. The hot flash data was collected retrospectively. Consequently, we are unable to completely exclude recall-bias concerning the grade and frequency of the hot flashes. Also, the modest sample size of this retrospective study requires that these results should be interpreted with care. Furthermore, only a single questionnaire was completed per patient, and as such we are unable to identify fluctuations in frequency and severity of hot flashes over the course of the tamoxifen treatment period. To adjust for any potential confounding, the duration of tamoxifen treatment was included as a covariate in the analyses. Finally, we have insufficient data concerning co-medication, other than medication to relieve hot flashes, to include this factor in our analyses, however, in the ATAC analyses medication use was not found to be an independent predictor [12].

This is the first study reporting a difference within post-menopausal patients based on their pre-treatment hot flash history in the association between tamoxifen and its main metabolite serum concentrations and hot flash frequency. This possible effect should be investigated further and requires validation in other series.

As we are unable to show that hot flash assessments are unambiguously indicative for therapeutic serum concentrations of endoxifen, and given that the value of pharmacogenomics is currently under debate, we think that future research could focus on measurement of active metabolite concentrations as a potential surrogate biomarker for tamoxifen efficacy.

## Conclusions

We are unable to confirm positive associations between active tamoxifen metabolite concentrations and either the frequency or severity of hot flashes during tamoxifen treatment, when ignoring menopausal status and pre-treatment hot flash history. However, within the post-menopausal women experiencing hot flashes prior to treatment, there is evidence for positive associations between serum concentrations of tamoxifen and its metabolites with hot flash frequency.

## Competing interests

The authors declare that they have no competing interests.

## Authors’ contributions

JHB, SCL, ADRH, RHTK and NGLJ designed the study. RHTK and NGLJ handled the questionnaires. NGLJ conducted the analysis of tamoxifen and its metabolites, TMK the DNA isolation and the estradiol measurements and RHNS the genotyping. ADV performed the statistical analyses. RHTK and NGLJ mainly wrote the manuscript. All authors read and approved the manuscript.

## Pre-publication history

The pre-publication history for this paper can be accessed here:

http://www.biomedcentral.com/1471-2407/13/612/prepub

## Supplementary Material

Additional file 1 Questionnaire. Click here for file

Additional file 2 Correlations between age, estradiol level, BMI, tamoxifen and its main metabolites. Click here for file

Additional file 3 Association between tamoxifen, its metabolites and estradiol concentrations and CYP2D6 genotype predicted phenotype. Click here for file

Additional file 4 Mean concentrations of tamoxifen, its metabolites and estradiol categorized by hot flash frequency and hot flash severity. Click here for file

Additional file 5 Multivariable regressions estimates of hot flash frequency (S5A) and severity (S5B) for each of the five factors of primary interest, adjusting for age, log transformed estradiol concentration, BMI, duration of treatment, menopausal status and pre-treatment hot-flash history. Click here for file

Additional file 6 Patient characteristics by menopausal status and pretreatment or no-pretreatment hot flashes. Click here for file
