# Supplementary material for: Hot flashes are not predictive for serum concentrations of tamoxifen and its metabolites
Source: BMC Cancer. 2013 Dec 28;13:612. doi: 10.1186/1471-2407-13-612 (PMC3880169; doi:10.1186/1471-2407-13-612)
Supplement: Additional file 1 — Questionnaire. [file 1471-2407-13-612-S1.docx]

**Additional file 1: S1** Questionnaire **Attachment – definitions of hot flashes**For describing the severity of your hot flashes, you can use the examples described below, formulated by cancer patients in previous studies. These formulations can help you describe your hot flashes as mild, moderate, severe or very severe.

MILD

*Time:* to 5 minutes

*Physical symptoms:* warmth, felt uncomfortable, red face

*Emotional symptoms:* not expected

*Action:* none

MODERATE

*Time:* to 15 minutes

*Physical symptoms:* head, neck, ears or whole body feels warm; tense, tight muscles, clammy (wet skin); a change in heart rate or rhythm (heart speeds up or changes beat); some sweating, dry mouth

*Emotional symptoms:* felt irritated, felt agitated (restless), felt as though energy was drained out, felt embarrassed when having a hot flash in front of others, felt tired, felt annoyed

*Action:* needed to use a fan, awakened sometimes at night, needed to uncover, took off layers of clothing, drank water, opened the windows even when cold outside, wore lighter clothing

SEVERE

*Time:* to 20 minutes

*Physical symptoms:* warmth, like a burning oven, a change in heart rate or rhythm (heart speeds up or changes beat); felt faint; headache; severe sweating; weakness, a pricking, stinging sensation over skin; chest heavyness

*Emotional symptoms:* embarrassment, anxiety, feelings of having a panic attack

*Action:* needed to stop what was being done at the time, waking up at night and removed covers, needed to remove clothes, opened windows, kept the house a cooler temperature, frequently used fans

VERY SEVERE

*Time:* to 45 minutes

*Physical symptoms:* boiling heat, rolling sweat, difficult breathing, felt faint, felt dizzy, feet and/or legs cramping, a change in heart rate or rhythm (heart speeds up or changes beat); felt slightly sick to stomach

*Emotional symptoms:* felt distressed, had the urge to escape, had difficulty functioning

*Action:* awakened frequently at night, needed to change sheets and pajamas, needed to take a cold shower, needed to hold ice on the skin
