# Supplementary material for: Hot flashes are not predictive for serum concentrations of tamoxifen and its metabolites
Source: BMC Cancer. 2013 Dec 28;13:612. doi: 10.1186/1471-2407-13-612 (PMC3880169; doi:10.1186/1471-2407-13-612)
Supplement: Additional file 2 — Correlations between age, estradiol level, BMI, tamoxifen and its main metabolites. [file 1471-2407-13-612-S2.docx]

**Additional file 2. S2** Correlations between age, estradiol level, BMI, tamoxifen and its main metabolites.

**Table S2a** Spearman’s correlation.

|  | **Age** | **Estradiol level** | **BMI** | **Tamoxifen** | **ND-tamoxifen** | **Endoxifen** |
| --- | --- | --- | --- | --- | --- | --- |
| Estradiol level | -0.27 |  |  |  |  |  |
| BMI | 0.05 | -0.02 |  |  |  |  |
| Tamoxifen | 0.13 | 0.01 | 0.07 |  |  |  |
| ND-tamoxifen | 0.00 | 0.03 | -0.12 | 0.83 |  |  |
| Endoxifen | 0.07 | 0.05 | 0.01 | 0.46 | 0.14 |  |
| 4OH-tamoxifen | 0.05 | 0.14 | 0.00 | 0.69 | 0.43 | 0.82 |

**Table S2b** P-values for test of Spearman’s correlation being different from zero.

|  | **Age** | **Estradiol level** | **BMI** | **Tamoxifen** | **ND-tamoxifen** | **Endoxifen** |
| --- | --- | --- | --- | --- | --- | --- |
| Estradiol level | 0.01 |  |  |  |  |  |
| BMI | 0.64 | 0.86 |  |  |  |  |
| Tamoxifen | 0.19 | 0.93 | 0.50 |  |  |  |
| ND-tamoxifen | 1.00 | 0.75 | 0.21 | <0.0001 |  |  |
| Endoxifen | 0.46 | 0.64 | 0.93 | <0.0001 | 0.15 |  |
| 4OH-tamoxifen | 0.63 | 0.16 | 1.00 | <0.0001 | <0.0001 | <0.0001 |

ND-tamoxifen: N-desmethyltamoxifen. 4OH-tamoxifen: 4-hydroxytamoxifen.
