# Supplementary material for: Hot flashes are not predictive for serum concentrations of tamoxifen and its metabolites
Source: BMC Cancer. 2013 Dec 28;13:612. doi: 10.1186/1471-2407-13-612 (PMC3880169; doi:10.1186/1471-2407-13-612)
Supplement: Additional file 3 — Association between tamoxifen, its metabolites and estradiol concentrations and CYP2D6 genotype predicted phenotype. [file 1471-2407-13-612-S3.docx]

|  | **CYP2D6 genotype predicted phenotype** | | |
| --- | --- | --- | --- |
|  | **EM**  **n=54** | **IM**  **n=30** | **PM**  **n=5** |
| Mean tamoxifen concentration (ng/mL) | 99.0 | 104 | 109 |
| Range | 39.7 - 237 | 50.0 - 220 | 59.2 - 219 |
| Mean N-desmethyltamoxifen concentration (ng/mL) | 171 | 227 | 252 |
| Range | 82.3 - 335 | 94.7 - 532 | 155 - 439 |
| Mean endoxifen concentration (ng/mL) | 11.3 | 7.11 | 3.06 |
| Range | 4.01 – 22.6 | 2.68 – 14.6 | 1.73 – 5.80 |
| Mean 4-hydroxytamoxifen concentration (ng/mL) | 1.86 | 1.71 | 1.36 |
| Range | 0.78 – 3.90 | 0.84 – 4.23 | 0.74 – 2.67 |
| Mean estradiol concentration (pmol/L) | 68.8 | 374 | 37.4 |
| Range | <LLOQ - 883 | <LLOQ - 3688 | <LLOQ – 67.0 |

**Additional file 3. S3** Association between tamoxifen, its metabolites and estradiol concentrations and CYP2D6 genotype predicted phenotype
