# Supplementary material for: Hot flashes are not predictive for serum concentrations of tamoxifen and its metabolites
Source: BMC Cancer. 2013 Dec 28;13:612. doi: 10.1186/1471-2407-13-612 (PMC3880169; doi:10.1186/1471-2407-13-612)
Supplement: Additional file 4 — Mean concentrations of tamoxifen, its metabolites and estradiol categorized by hot flash frequency and hot flash severity. [file 1471-2407-13-612-S4.docx]

|  | **Frequency of hot flashes per week** | | | | |
| --- | --- | --- | --- | --- | --- |
|  | **0 (n=17)** | **1-20 (n=24)** | **21-50 (=41)** | **51-100 (n=18)** | **>100 (n=9)** |
| Tamoxifen concentration (ng/mL) | | | | | |
| Mean (SD) | 103 (32.1) | 113 (49.8) | 100 (35.7) | 96.6 (35.6) | 120 (53.0) |
| Median (range) | 103 (57.2-161) | 97.5 (52.6-220) | 93.0 (39.7-219) | 92.7 (50.0-155) | 107 (72.5-237) |
| N-desmethyltamoxifen concentration (ng/mL) | | | | | |
| Mean (SD) | 203 (57.3) | 209 (88.2) | 200 (86.2) | 180 (76.3) | 214 (47.1) |
| Median (range) | 196 (115-301) | 191 (94.7-388) | 177 (82.3-532) | 156 (92.6-341) | 212 (159-285) |
| Endoxifen concentration (ng/mL) | | | | | |
| Mean (SD) | 9.28 (5.59) | 10.4 (4.43) | 9.78 (4.81) | 8.24 (4.44) | 9.40 (5.22) |
| Median (range) | 7.67 (1.93-22.6) | 10.1 (2.68-17.7) | 9.62 (2.14-22.1) | 8.19 (1.73-18.6) | 7.24 (3.18-16.7) |
| 4-hydroxytamoxifen concentration (ng/mL) | | | | | |
| Mean (SD) | 1.86 (0.84) | 2.01 (0.75) | 1.77 (0.72) | 1.61 (0.53) | 1.91 (0.85) |
| Median (range) | 1.81 (0.74-3.90) | 1.87 (0.84-3.51) | 1.60 (0.78-4.23) | 1.47 (0.87-2.70) | 1.54 (1.11-3.29) |
| Estradiol concentration (pmol/L) | | | | | |
| Mean (SD) | 434 (824) | 324 (807) | <LLOQ (29.5) | 143 (272) | 121 (271) |
| Median (range) | <LLOQ  (<LLOQ-3012) | 35  (<LLOQ-3688) | <LLOQ  (<LLOQ-142) | <LLOQ  (<LLOQ-839) | <LLOQ  (<LLOQ-844) |

**Additional file 4: S4** Mean concentrations of tamoxifen, its metabolites and estradiol categorized by hot flash frequency and hot flash severity

**Table S4a** Mean concentrations of tamoxifen, its metabolites and estradiol categorized by hot flash frequency

**Table S4b** Mean concentrations of tamoxifen, its metabolites and estradiol categorized by hot flash severity

|  | **Frequency of hot flashes per week** | | | | |
| --- | --- | --- | --- | --- | --- |
|  | **0 (n=17)** | **1-20 (n=24)** | **21-50 (=41)** | **51-100 (n=18)** | **>100 (n=9)** |
| Tamoxifen concentration (ng/mL) | | | | | |
| Mean (SD) | 103 (32.1) | 113 (49.8) | 100 (35.7) | 96.6 (35.6) | 120 (53.0) |
| Median (range) | 103 (57.2-161) | 97.5 (52.6-220) | 93.0 (39.7-219) | 92.7 (50.0-155) | 107 (72.5-237) |
| N-desmethyltamoxifen concentration (ng/mL) | | | | | |
| Mean (SD) | 203 (57.3) | 209 (88.2) | 200 (86.2) | 180 (76.3) | 214 (47.1) |
| Median (range) | 196 (115-301) | 191 (94.7-388) | 177 (82.3-532) | 156 (92.6-341) | 212 (159-285) |
| *(Z)-*endoxifen concentration (ng/mL) | | | | | |
| Mean (SD) | 9.28 (5.59) | 10.4 (4.43) | 9.78 (4.81) | 8.24 (4.44) | 9.40 (5.22) |
| Median (range) | 7.67 (1.93-22.6) | 10.1 (2.68-17.7) | 9.62 (2.14-22.1) | 8.19 (1.73-18.6) | 7.24 (3.18-16.7) |
| 4-hydroxytamoxifen concentration (ng/mL) | | | | | |
| Mean (SD) | 1.86 (0.839) | 2.01 (0.752) | 1.77 (0.719) | 1.61 (0.527) | 1.91 (0.845) |
| Median (range) | 1.81 (0.74-3.90) | 1.87 (0.84-3.51) | 1.60 (0.78-4.23) | 1.47 (0.87-2.70) | 1.54 (1.11-3.29) |
| Estradiol concentration (pmol/L) | | | | | |
| Mean (SD) | 434 (824) | 324 (807) | <LLOQ (29.5) | 143 (272) | 121 (271) |
| Median (range) | <LLOQ  (<LLOQ-3012) | 35  (<LLOQ-3688) | <LLOQ  (<LLOQ-142) | <LLOQ  (<LLOQ-839) | <LLOQ  (<LLOQ-844) |

|  | | **Average severity of hot flashes** | | | | | | | | |
| --- | --- | --- | --- | --- | --- | --- | --- | --- | --- | --- |
|  | | **None**  **(n=17)** | | **Mild**  **(n=22)** | | **Moderate (n=55)** | | **Severe (n=11)** | | **Very severe (n=4)** |
| Tamoxifen concentration (ng/mL) | | | | | | | | | | |
| Mean (SD) | 103 (32.1) | | 109 (45.0) | | 98.3 (36.1) | | 126 (58.2) | | 112 (32.7) | |
| Median (range) | 103 (57.2-161) | | 92.4 (50.3-197) | | 94.5 (49.3-219) | | 121 (39.7-237) | | 104 (85.1-156) | |
| N-desmethyltamoxifen concentration (ng/mL) | | | | | | | | | | |
| Mean (SD) | 203 (57.3) | | 210 (110) | | 193 (70.3) | | 203 (80.2) | | 231 (50.0) | |
| Median (range) | 196 (115-301) | | 170 (94.3-532) | | 180 (92.6-439) | | 181 (82.3-388) | | 234 (171-285) | |
| Endoxifen concentration (ng/mL) | | | | | | | | | | |
| Mean (SD) | 9.28 (5.59) | | 10.1 (3.44) | | 9.58 (5.02) | | 9.43 (4.95) | | 7.81 (6.01) | |
| Median (range) | 7.67 (1.93-22.6) | | 10.2 (4.16-16.8) | | 8.84 (2.14-22.0) | | 9.27 (1.73-18.0) | | 5.26 (4.01-16.7) | |
| 4-hydroxytamoxifen concentration (ng/mL) | | | | | | | | | | |
| Mean (SD) | 1.86 (0.84) | | 1.99 (0.82) | | 1.73 (0.63) | | 1.91 (0.74) | | 1.67 (1.09) | |
| Median (range) | 1.81 (0.74-3.90) | | 1.87 (0.89-4.23) | | 1.54 (0.84-3.23) | | 2.01 (0.78-2.96) | | 1.25 (0.90-3.29) | |
| Estradiol concentration (pmol/L) | | | | | | | | | | |
| Mean (SD) | 434 (824) | | 313 (835) | | 106 (246) | | 43.5 (32.8) | | <LLOQ (-) | |
| Median (range) | <LLOQ  (<LLOQ-3012) | | <LLOQ  (<LLOQ-3688) | | <LLOQ  (<LLOQ-1420) | | <LLOQ  (<LLOQ-119) | | <LLOQ  (<LLOQ) | |

SD: standard deviation. LLOQ: lower limit of quantification.
