# Supplementary material for: Hot flashes are not predictive for serum concentrations of tamoxifen and its metabolites
Source: BMC Cancer. 2013 Dec 28;13:612. doi: 10.1186/1471-2407-13-612 (PMC3880169; doi:10.1186/1471-2407-13-612)
Supplement: Additional file 5 — Multivariable regressions estimates of hot flash frequency (S5A) and severity (S5B) for each of the five factors of primary interest, adjusting for age, log transformed estradiol concentration, BMI, duration of treatment, menopausal status and pre-treatment hot-flash history. [file 1471-2407-13-612-S5.docx]

**Additional file 5: S5** Multivariable regressions estimates of hot flash frequency (S5A) and severity (S5B) for each of the five factors of primary interest, adjusting for age, log transformed estradiol concentration, BMI, duration of treatment, menopausal status and pre-treatment hot-flash history. In the models in table S5A the interactions between the respective factor of interest and menopause and pre-treatment history are also included.

**S5A** Associations with hot flush frequency.

|  |  | **Coef** | **SE** | **p-value** | **p-value** |
| --- | --- | --- | --- | --- | --- |
| **Tamoxifen** | |  |  |  |  |
|  | Intercept (pre-M) | 4.2 | 0.59 | <0.0001 |  |
|  | post-M & PTHF v pre-M | 0.074 | 0.32 | 0.82 |  |
|  | post-M & no PTHF v pre-M | -0.18 | 0.29 | 0.55 |  |
|  | Age | -0.01 | 0.013 | 0.42 |  |
|  | Estradiol concentration | -0.12 | 0.098 | 0.24 |  |
|  | BMI | 0.06 | 0.024 | 0.01 |  |
|  | Tamoxifen duration | 0.081 | 0.10 | 0.42 |  |
|  | CYP2D6 phenotype | -0.31 | 0.22 | 0.15 |  |
|  | Tam:pre-M | -0.0057 | 0.0043 | 0.19 | 0.02 |
|  | Tam:post-M & PTHF v pre-M | 0.020 | 0.008 | 0.01 |  |
|  | Tam:post-M & no PTHF v pre-M | 0.0095 | 0.0055 | 0.09 |  |
| **N-desmethyltamoxifen** | |  |  |  |  |
|  | Intercept (pre-M) | 4.2 | 0.6 | <0.0001 |  |
|  | post-M & PTHF v pre-M | 0.057 | 0.34 | 0.87 |  |
|  | post-M & no PTHF v pre-M | -0.0054 | 0.29 | 0.99 |  |
|  | Age | -0.0095 | 0.013 | 0.47 |  |
|  | Estradiol concentration | -0.13 | 0.10 | 0.22 |  |
|  | BMI | 0.052 | 0.025 | 0.04 |  |
|  | Tamoxifen duration | 0.11 | 0.10 | 0.30 |  |
|  | CYP2D6 phenotype | -0.42 | 0.23 | 0.08 |  |
|  | ND-tam:pre-M | -0.0022 | 0.0023 | 0.34 | 0.05 |
|  | ND-tam:post-M & PTHF v pre-M | 0.0086 | 0.0039 | 0.03 |  |
|  | ND-tam:post-M & no PTHF v pre-M | -0.00049 | 0.0033 | 0.88 |  |
| **Endoxifen** | |  |  |  |  |
|  | Intercept (pre-M) | 4.3 | 0.6 | <0.0001 |  |
|  | post-M & PTHF v pre-M | 0.27 | 0.32 | 0.41 |  |
|  | post-M & no PTHF v pre-M | -0.0013 | 0.28 | 1.00 |  |
|  | Age | -0.014 | 0.013 | 0.28 |  |
|  | Estradiol concentration | -0.12 | 0.097 | 0.20 |  |
|  | BMI | 0.056 | 0.024 | 0.02 |  |
|  | Tamoxifen duration | 0.021 | 0.087 | 0.81 |  |
|  | CYP2D6 phenotype | -0.26 | 0.24 | 0.28 |  |
|  | End.:pre-M | -0.048 | 0.036 | 0.18 | 0.02 |
|  | End.:post-M & PTHF v pre-M | 0.15 | 0.054 | 0.01 |  |
|  | End.:post-M & no PTHF v pre-M | 0.05 | 0.053 | 0.35 |  |
| **4-Hydroxytamoxifen** | |  |  |  |  |
|  | Intercept (pre-M) | 4.3 | 0.6 | <0.0001 |  |
|  | post-M & PTHF v pre-M | 0.18 | 0.32 | 0.57 |  |
|  | post-M & no PTHF v pre-M | -0.024 | 0.28 | 0.93 |  |
|  | Age | -0.013 | 0.013 | 0.32 |  |
|  | Estradiol concentration | -0.12 | 0.099 | 0.22 |  |
|  | BMI | 0.058 | 0.025 | 0.02 |  |
|  | Tamoxifen duration | 0.02 | 0.093 | 0.83 |  |
|  | CYP2D6 phenotype | -0.34 | 0.22 | 0.13 |  |
|  | 4OH-tam:pre-M | -0.20 | 0.22 | 0.34 | 0.02 |
|  | 4OH-tam:post-M & PTHF v pre-M | 0.96 | 0.35 | 0.01 |  |
|  | 4OH-tam:post-M & no PTHF v pre-M | 0.12 | 0.38 | 0.76 |  |

*Interaction p-value.

Coef = coefficient; SE = standard error; BMI = body mass index; post-M = post-menopausal; pre-M = pre-menopausal; PTHF = pre-treatment hot flashes; Tam = tamoxifen; ND-tam = N-desmethyltamoxifen End = endoxifen; 4OH-tam = 4-Hydroxytamoxifen.

**S5B** Associations with hot flush severity. P-value for the menopause & pre-treatment hot flash status is for two degrees of freedom.

|  |  | **Coef** | **SE** | **p-value** |
| --- | --- | --- | --- | --- |
| **Tamoxifen** | |  |  |  |
|  | Age | -0.054 | 0.025 | 0.03 |
|  | Estradiol concentration | -0.46 | 0.16 | 0.003 |
|  | BMI | 0.21 | 0.05 | <0.0001 |
|  | Tamoxifen duration | 0.004 | 0.21 | 0.98 |
|  | CYP2D6: I/PM v EM | -0.42 | 0.40 | 0.29 |
|  | post-M & PTHF v pre-M | 1.5 | 0.6 | 0.02 |
|  | post-M & no PTHF v pre-M | 0.004 | 0.51 |  |
|  | Tamoxifen | 0.0064 | 0.0051 | 0.21 |
| **N-desmethyltamoxifen** | | |  |  |
|  | Age | -0.049 | 0.025 | 0.05 |
|  | Estradiol concentration | -0.44 | 0.16 | 0.004 |
|  | BMI | 0.21 | 0.05 | <0.0001 |
|  | Tamoxifen duration | 0.06 | 0.21 | 0.77 |
|  | CYP2D6: I/PM v EM | -0.36 | 0.42 | 0.39 |
|  | post-M & PTHF v pre-M | 1.40 | 0.62 | 0.03 |
|  | post-M & no PTHF v pre-M | -0.03 | 0.51 |  |
|  | N-desmethyltamoxifen | 0.0017 | 0.0026 | 0.52 |
| **Endoxifen** | |  |  |  |
|  | Age | -0.049 | 0.025 | 0.04 |
|  | Estradiol concentration | -0.44 | 0.15 | 0.004 |
|  | BMI | 0.21 | 0.05 | <0.0001 |
|  | Tamoxifen duration | 0.076 | 0.20 | 0.71 |
|  | CYP2D6: I/PM v EM | -0.65 | 0.46 | 0.16 |
|  | post-M & PTHF v pre-M | 1.5 | 0.6 | 0.02 |
|  | post-M & no PTHF v pre-M | -0.019 | 0.51 |  |
|  | Endoxifen | 0.048 | 0.047 | 0.31 |
| **4-Hydroxytamoxifen** | | |  |  |
|  | Age | -0.049 | 0.025 | 0.05 |
|  | Estradiol concentration | -0.43 | 0.16 | 0.004 |
|  | BMI | 0.21 | 0.051 | <0.0001 |
|  | Tamoxifen duration | 0.081 | 0.21 | 0.70 |
|  | CYP2D6: I/PM v EM | -0.45 | 0.41 | 0.27 |
|  | post-M & PTHF v pre-M | 1.4 | 0.6 | 0.03 |
|  | post-M & no PTHF v pre-M | 0.01 | 0.51 |  |
|  | 4-Hydroxytamoxifen | 0.13 | 0.29 | 0.65 |

Coef = coefficient; SE = standard error; BMI = body mass index; post-M = post-menopausal; pre-M = pre-menopausal; PTHF = pre-treatment hot flashes.
