# Supplementary material for: Hot flashes are not predictive for serum concentrations of tamoxifen and its metabolites
Source: BMC Cancer. 2013 Dec 28;13:612. doi: 10.1186/1471-2407-13-612 (PMC3880169; doi:10.1186/1471-2407-13-612)
Supplement: Additional file 6 — Patient characteristics by menopausal status and pretreatment or no-pretreatment hot flashes. [file 1471-2407-13-612-S6.docx]

**Additional file 6: S6** Patient characteristics by menopausal status and pretreatment or no-pretreatment hot flashes

**S6a** Baseline patient characteristics

| Characteristics | **Total** **cohort** | **Pre-**  **menopausal** | **Post-menopausal with PTHF** | **Post-menopausal no-PTHF** |
| --- | --- | --- | --- | --- |
|  | **n = 109**  *n (%)* | **n = 56**  *n (%)* | **n = 18**  *n (%)* | **N= 35**  *n (%)* |
|  |  |  |  |  |
| Median age at assessment (years) | 51 | 45 | 56 | 59 |
| Range | 22 – 76 | 22 – 54 | 42 – 71 | 40 – 76 |
|  |  |  |  |  |
| Median Body Mass Index | 24 | 24 | 24 | 25 |
| Range | 17 – 43 | 17 – 34 | 19 – 29 | 19 – 43 |
|  |  |  |  |  |
| T-status (TNM) |  |  |  |  |
| T1 | 48 (44%) | 23(41%) | 7 (39%) | 18 (51%) |
| T2 | 27 (25%) | 16 (29%) | 5 (28%) | 6 (17%) |
| T3 | 2 (1.8%) | 1 (1.8%) | 0 (0%) | 1 (3%) |
| Unknown | 32 (29%) | 16 (29%) | 6 (33%) | 10 (29%) |
|  |  |  |  |  |
| N-status (TNM) |  |  |  |  |
| N0 | 45 (41%) | 24 (43%) | 9 (50%) | 12 (34%) |
| N+ | 49 (45%) | 26 (46%) | 6 (33%) | 17 (49%) |
| Unknown | 15 (14%) | 6 (11%) | 3 (17%) | 6 (17%) |
|  |  |  |  |  |
| AJCC stage (7^th^ ed.) |  |  |  |  |
| Stage I | 26 (23%) | 14 (25%) | 4 (22%) | 8 (23%) |
| Stage IIa | 32 (29%) | 16 (29%) | 6 (33%) | 10 (29%) |
| Stage IIb  St | 7 (6%) | 5 (9%) | 1 (6%) | 1 (3%) |
| Stage IIIa | 9 (8%) | 4 (7%) | 1 (6%) | 4 (11%) |
| Stage IIIb | 0 | 0 | 0 | 0 |
| Stage IIIc | 6 (6%) | 3 (5%) | 1 (6%) | 2 (6%) |
| Unknown | 29 (29%) | 14 (25%) | 5 (28%) | 10 (29%) |
|  |  |  |  |  |
| Estrogen receptor |  |  |  |  |
| Positive | 93 (85%) | 50 (89%) | 14 (78%) | 29 (83%) |
| Unknown | 16 (15%) | 6 (11%) | 4 (22%) | 6 (17%) |
|  |  |  |  |  |
| Progesterone receptor |  |  |  |  |
| Positive | 68 (62%) | 37 (66%) | 10 (56%) | 21 (60%) |
| Negative | 24 (22%) | 13 (23%) | 4 (22%) | 7 (20%) |
| Unknown | 17 (16%) | 6 (11%) | 4 (22%) | 7 (20%) |
|  |  |  |  |  |
| HER2 status |  |  |  |  |
| Positive | 10 (9%) | 5 (9%) | 2 (11%) | 3 (9%) |
| Negative | 83 (76%) | 45 (80%) | 12 (34%) | 26 (74%) |
| Unknown | 16 (15%) | 6 (11%) | 4 (22%) | 6 (17%) |
|  |  |  |  |  |
| Median duration of treatment (months)  (months) | 9 | 9 | 9 | 9 |
| Range | 2 - 70 | 2 - 59 | 3 - 70 | 3 - 70 |
| Tamoxifen (daily dose) |  |  |  |  |
| 10 mg | 1 (1%) | 1 (2%) | 0 (0%) | 0 (0%) |
| 20 mg | 102 (94%) | 50(89%) | 18 (100%) | 34 (98%) |
| 40 mg | 6 (6%) | 5 (9%) | 0 (0%) | 1 (2%) |

|  | **Total** | **Pre-**  **menopausal** | **Post-menopausal with PTHF** | **Post-menopausal no PTHF** |
| --- | --- | --- | --- | --- |
|  | **n = 109**  *n (%)* | **n = 56**  *n (%)* | **n = 18**  *n (%)* | **n = 35**  *n (%)* |
|  |  |  |  |  |
| Pre-treatment history of hot flashes |  |  |  |  |
| No | 82 (75%) | 47 (84%) | 0 (0%) | 35 (100%) |
| Yes | 27 (25%) | 9 (16%) | 18 (100%) | 0 (0%) |
| Median frequency of hot flashes per week | 21 | 21 | 28 | 21 |
| Range | 0 - 168 | 0 - 168 | 4 - 112 | 0 - 168 |
|  |  |  |  |  |
| Average severity of hot flashes |  |  |  |  |
| None | 17 (16%) | 9 (16%) | 0 (0%) | 8 (23%) |
| Mild | 22 (20%) | 13 (23%) | 3 (17%) | 6 (17%) |
| Moderate | 55 (50%) | 26 (46%) | 11 (61%) | 18 (51%) |
| Severe | 11 (10%) | 7 (12%) | 2 (11%) | 2 (6%) |
| Very severe | 4 (4%) | 1 (2%) | 2 (11%) | 1 (3%) |
|  |  |  |  |  |
|  |  |  |  |  |
| Median tamoxifen (ng/mL) | 95.4 | 93.8 | 96.4 | 112 |
| Range | 39.7 - 237 | 50 - 220 | 39.7 - 156 | 49.3 - 237 |
|  |  |  |  |  |
| Median N-desmethyltamoxifen (ng/mL) | 181 | 177 | 189 | 208 |
| Range | 82.3 - 532 | 94.3 - 532 532 | 82.3 - 326 | 92.6 - 439 |
|  |  |  |  |  |
| Median endoxifen (ng/mL) | 9.12 | 8.59 | 8.40 | 10.5 |
| Range | 1.73 – 22.6 | 1.73 – 20.3 | 2.10 – 17.3 | 3.00 – 22.6 |
|  |  |  |  |  |
| Median 4-hydroxytamoxifen (ng/mL) | 1.69 | 1.77 | 1.70 | 1.90 |
| Range | 0.74 - 4.23 | 0.74 - 4.23 | 0.78 - 3.31 | 0.90 - 3.91 |
|  |  |  |  |  |
| Median estradiol (pmol/L)* |  |  |  |  |
| <LLOQ | 70 | 33 | 13 | 24 |
| 43.0 – 67.0 | 12 | 6 | 2 | 4 |
| 67.0 – 361 | 14 | 8 | 3 | 3 |
| >361 | 11 | 9 | 0 | 2 |
| Missing | 2 | 0 | 0 | 2 |
|  |  |  |  |  |
| CYP2D6 phenotype |  |  |  |  |
| Extensive metabolizer | 54 (50%) | 28 (50%) | 7 (39%) | 19 (54%) |
| Intermediate metabolizer | 30 (28%) | 19 (34%) | 3 (9%) | 8 (23%) |
| Poor metabolizer | 5 (4%) | 2 (4%) | 2 (11%) | 1 (3%) |
| Missing | 20 (18%) | 7 (12%) | 6 (33%) | 7 (20%) |

**S6B** Hot flash frequency and severity and pharmacological and biomedical parameters of study participants during treatment with tamoxifen, divided by menopausal status and pretreatment or no-pretreatment hot flashes
